# Changes of intraocular pressure after cataract surgery in myopic and emmetropic patients

**DOI:** 10.1097/MD.0000000000012023

**Published:** 2018-09-21

**Authors:** Huibin Lv, Jiarui Yang, Yushi Liu, Xiaodan Jiang, Yan Liu, Mingzhou Zhang, Yuexin Wang, Hang Song, Xuemin Li

**Affiliations:** Department of Ophthalmology, Peking University Third Hospital, Beijing, China.

**Keywords:** cataract surgery, glaucoma, intraocular pressure, myopia

## Abstract

To investigate the intraocular pressure (IOP) changes after cataract surgery, and its relationship with refractive conditions.

IOP after phacoemulsification with intraocular lens (IOL) implantation was retrospectively reviewed. Patients were classified into 3 groups by refractive conditions: emmetropia, mild to moderate myopia, and high myopia. Basic information was collected including age, sex, place of IOL, and operating surgeon, with IOP and refractive conditions measured before surgery, and 1, 7, 30, and 90 days after surgery.

The study comprised 353 eyes from 353 patients, of which 175 were emmetropia, 130 were mild to moderate myopia, and 48 were high myopia. A lower IOP than baseline was observed at 7, 30, and 90 days after surgery in emmetropic and mild to moderate myopia, while in high myopia, IOP was instable from 1 to 30 days, and reduced only in 90 days after surgery. Changes of IOP was more significant from 1 to 7 days in emmetropic and mild to moderate myopic patients, but from 30 to 90 days in high myopia. Patients over 75 showed a lower IOP at each follow-up than patients younger and female showed a higher baseline IOP than male. Different surgeons might influence the IOP fluctuation at first 90 days but not the final IOP.

All patients with different refractive conditions showed a remarkably lower IOP at 90 days after cataract surgery. However, high myopia lowered the speed of IOP reduction, which might be explained by the anatomical changes of eye structure.

## 1 Introduction

Cataract is identified as a leading cause of blindness worldwide,^[1]^ and the prevalence of cataract has been increasing in recent years as incidence of cataract is age-related^[2]^ and the aging of population has become a global problem.^[3]^ Cataract surgery has been performed for years and was proved to be valuable in visual improvement.^[4]^ Numerous studies have reported that cataract surgery played a key role in the reduction of intraocular pressure (IOP) in both glaucoma and nonglaucoma patients,^[5]^ and a recent study revealed that surgery of cataract showed great efficacy, recommending cataract surgery to be a 1st-line treatment for patients with glaucoma.^[6]^ However, factors that might influence the reduction of IOP after cataract surgery remains to be elucidated.

Several factors influencing the IOP after cataract surgery have been explored in previous studies. Preoperative IOP value was considered a predictive factor of IOP after surgery in open-angle glaucoma,^[7]^ and anatomic changes like increase in anterior chamber depth, angle opening, and posterior capsule moving posteriorly have been reported to be the risk factors for IOP elevation.^[8,9,17]^

Myopia is the most common medical conditions of the eyes, and the prevalence of myopia has been increasing in recent years, especially in eastern Asia.^[10]^ Myopia, especially pathologic myopia, might induce anatomical changes like chorioretinal thinning with the elongation of visual axis, and further lead to some ophthalmic complications such as glaucoma and rhegmatogenous retinal detachment.^[11]^ Although a notable correlation between myopia and IOP elevation has long been identified,^[12,13]^ the role of myopia in IOP after cataract surgery remains unclear.

In this study, a retrospective cohort study was performed to assess the changes of IOP and the role of myopia in IOP after cataract surgery.

## 2 Methods

### 2.1 Study design and participants

This study was a retrospective cohort study conducted according to the principles of the Declaration of Helsinki and was approved by the Human Research and Ethics Committee of Peking University Third Hospital. All information was collected from the follow-up system of cataract surgery in Department of Ophthalmology.

The study included patients: with a diagnosis of age-related cataract; who finished preoperative examinations; receiving a phacoemulsification (phaco)/extracapsular cataract extraction (ECCE) and intraocular lens (IOL) implantation under topical anesthesia; and who participated in the follow-ups in the first 3 months after surgery. For this study, records of the following conditions were excluded in patients: with uncontrolled systemic disease and eye diseases other than cataract and refractive errors (especially glaucoma or inflammatory eye disease that might affect the IOP); who had intraocular surgery before the study; receiving general anesthesia during the surgery; and under the age of 18.

Eventually, 353 eyes were enrolled in this study.

### 2.2 Clinical evaluation

The clinical assessments of the enrolled subjects were carried out in following order: basic information collection (including age, sex, place of IOL, and operating surgeon), presurgery examinations (refractive conditions and IOP), and postsurgery examinations at each follow-up (refractive conditions and IOP).

#### 2.2.1 Evaluation of refractive conditions

The objective monocular refractive condition for the operating eye was measured before surgery, using Nidek Auto Ref/Keratometer ARK-730A (Nidek Co Ltd, Aichi, Japan), and the implanted IOL was determined by IOL-Master 500 (Carl Zeiss Meditec, Jena, Germany). As a result of the opacity of lens, no studies before have successfully evaluated refractive power in cataract patients, and the final refractive diopter in this study was determined following a 4-step procedure:

Patients’ history: for the long-term follow-up patients, an initial evaluation was conducted by the latest follow-up information of patients’ history in refractive conditions recorded in our follow-up system. And for newly diagnosed patients without follow-up records, the refractive conditions were evaluated mainly by the self-reported refractive history and the diopter of currently worn glasses. A refractive history of 0 to −0.25D was preliminary defined as emmetropia, −0.5 to −5.75 as mild to moderated myopia, and −6.0 and higher as high myopia.

All patients went through a thorough physical examination.

All patients went through Keratometer and IOL master evaluations.

Overall assessment: to avoid the possible influence of cataract condition on refractive diopter, patients with a mismatch of previous history and the examination results were excluded.

The final refractive conditions were determined by an experienced optometrist according to the results obtained during the procedure, dividing patients into 3 groups including emmetropia, mild to moderated myopia, and high myopia.

#### 2.2.2 IOP measurement

All patients received a topical anesthesia twice in both eyes with an interval of 5 minutes, then applied with sodium fluorescein, and IOP was taken as an average of 2 readings by Goldmann tonometry. The performer of these measurement was masked to the research program and refractive conditions of the patients. Data of IOP in the surgery eye was recorded in follow-up system of cataract surgery in Department of Ophthalmology, Peking University Third Hospital. IOP was measured at same time of the day before surgery, and at 1, 7, 30, and 90 days after surgery.

ΔIOP at each follow-up was defined as IOP at this follow-up minus IOP at last follow-up (e.g., ΔIOP at 30 days = IOP at 30 days–IOP at 7 days). Total IOP Change at each follow-up was defined as IOP at each follow-up minus IOP before surgery (e.g., Total IOP Change at 30 days = IOP at 30 days–IOP before surgery).

### 2.3 Statistical analysis

Statistical analyses were conducted using SPSS software version 22.0 (SPSS, Inc., Chicago, IL). For each analysis, grouped and over-all analyses were performed. Basic characteristics of the 3 groups were described using descriptive statistics including means, standard deviations for continuous variables, and frequencies and proportions for categorical variables. A one-way ANOVA was used to analyze the continuously numeric variables with a Dunnett post-hoc applied in comparison of each group, and Chi-square test was applied in classified variables. Paired *t* test was used to assess the differences of IOP at each follow-up from baseline along with ΔIOP in each refractive group. Furthermore, an independent *t* test was implemented to determine differences in IOP at each time point as well as Total IOP Changes between 2 groups, and a one-way ANOVA was applied between 3 refractive conditions. Finally, the same procedure mentioned above was employed after stratifying by age (≤75 and >75 years), gender, and operating surgeon, respectively. A *P* value less than .05 was considered significant.

## 3 Results

A total of 353 eyes of 353 patients who underwent cataract surgery at Peking University Third Hospital between the period of December 2016 and September 2017 and fit all of the inclusion criteria were enrolled, of which 175 were emmetropia, 130 were mild to moderate myopia, and 48 were high myopia. Patient demographics are shown in Table 1.

**Table 1:**
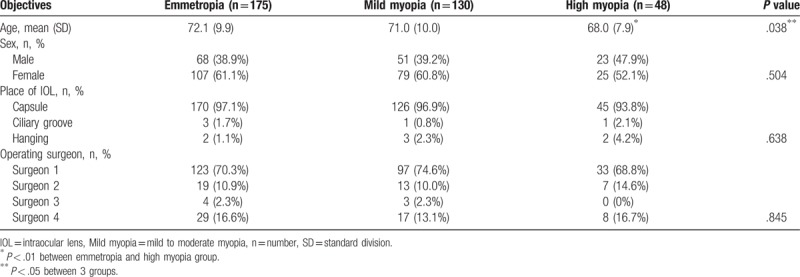
Basic characteristics of enrolled subjects.

Characteristics including sex, place of implanted IOL, and operating surgeon were matched between groups, with a significant younger age observed in high myopia group than emmetropia group (*P* = .011). Of these patients, 332 patients finished the follow-up at 1 day after surgery, 276 at 7 days, 190 at 30 days, and 86 at 90 days. The lost data mainly came from patients who were from areas other than Beijing and chose to finish the follow-up at local hospitals.

### 3.1 Differences of IOP at each follow-up

**Figure 1:**
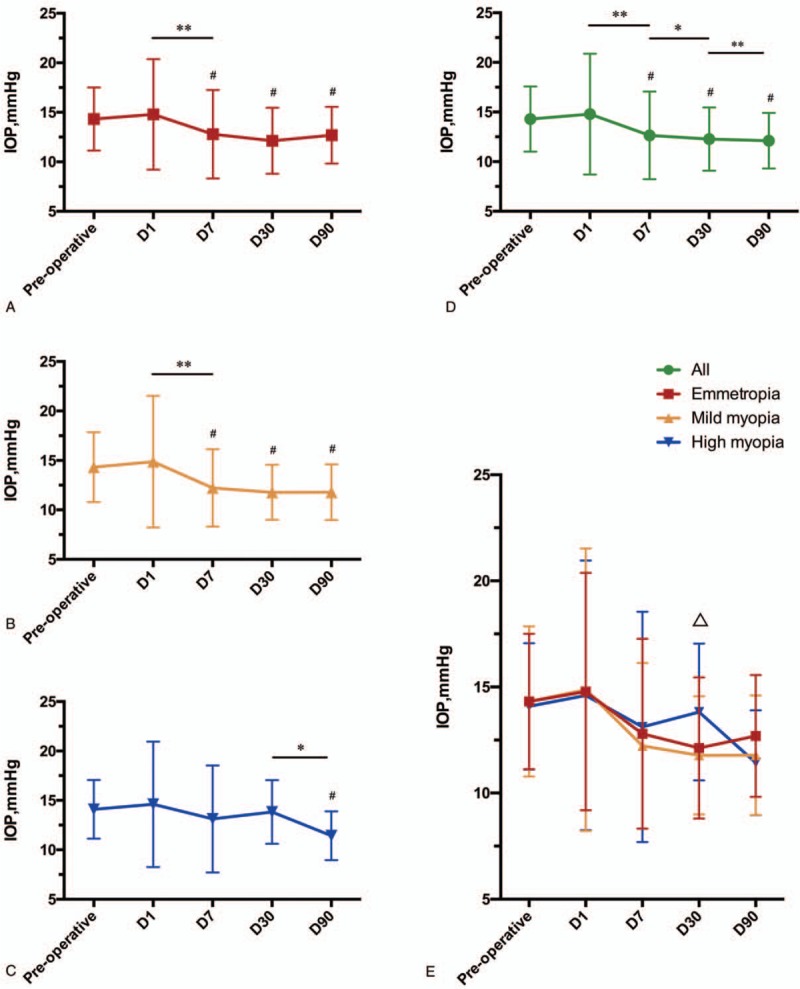
Different intraocular pressure at each follow-up before and after surgery. (A) IOP at each follow-up before and after surgery in emmetropia patients. (B) IOP at each follow-up before and after surgery in mild to moderate myopia patients. (C) IOP at each follow-up before and after surgery in high myopia patients. (D) IOP at each follow-up before and after surgery in all patients. (E) Comparison of IOP between emmetropia, mild to moderate myopia, and high myopia patients. Mild myopia, mild to moderate myopia; D, days after operation. *P* < .01 compared with IOP before surgery was marked with #. *P* < .05 compared with last follow-up was marked with ∗. *P* < .01 compared with last follow-up was marked with ∗∗. *P* < .05 compared between groups was marked with ^Δ^. IOP = intraocular pressure.

Figure 1 shows the IOP at each follow-up in all subjects and in 3 groups separately. Overall, IOP at 1 day after surgery (14.8 ± 6.1) was higher than IOP before surgery (14.3 ± 3.3), while it did not reach a statistical significance (*P* = .105). IOP at 7 days (12.6 ± 4.4), 30 days (12.2 ± 3.2), and 90 days (12.1 ± 2.8) was significantly lower than that before surgery (all with *P* < .001), and the mean IOP was significantly lower at 7 days compared with that at 1 day (*P* < .001), 30 days compared with 7 days (*P* = .029), and 90 days compared with 30 days (*P* = .005). These results indicated that IOP was decreasing with the elongation of time after surgery (Fig. 1D).

Emmetropic patients showed a higher IOP at 1 day after surgery (14.8 ± 5.6) than that before surgery (14.3 ± 3.2), while it did not reach a statistically significant difference (*P* = .294). IOP at 7 days (12.8 ± 4.5), 30 days (12.1 ± 3.3), and 90 days (12.7 ± 2.9) was significantly lower than that before surgery (all with *P* < .001), and the mean IOP was significantly lower at 7 days compare with 1 day (*P* < .001), IOP at 30 days was lower than that at 7 days, while it did not reach a significant difference (*P* = .061), and IOP at 90 days was higher compared with 30 days, which did not reach a statistically significant level either (*P* *=* .126) (Fig. 1A).

Myopic patients were subcategorized into mild to moderate myopia and severe myopia, and the results indicated that mild to moderate myopia showed a rather similar result to emmetropic patients, as IOP at 1 day (14.9 ± 6.7) was higher than IOP before surgery (14.3 ± 3.5) while it did not reach a statistically significant difference (*P* *=* .218), and IOP at 7 days (12.2 ± 3.9), 30 days (11.8 ± 2.8), and 90 days (11.8 ± 2.8) were significantly lower than that before surgery (all with *P* < .001). IOP was significantly lower at 7 days compared with 1 day (*P* < .001), IOP at 30 days was lower than that at 7 days, while it did not reach a significant difference (*P* *=* .103), and IOP at 90 days was slightly higher compared with 30 days with no statistical significant difference either (*P* *=* .173) (Fig. 1B).

Meanwhile, IOP in high myopia showed a higher IOP at 1 day (14.6 ± 6.4) and lower IOP at 7 days (13.1 ± 5.4) and 30 days (13.8 ± 3.2) compared with IOP before surgery (14.0 ± 3.0), while it did not reach a statistically significant level (*P* *=* .531, *P* *=* .305, and *P* *=* .053, respectively). IOP at 90 days (11.4 ± 2.5) was significantly lower than IOP before surgery (*P* < .001). IOP at 7 days compared with 1 day (*P* *=* .180), 30 days compared with 7 days (*P* *=* .666) showed no significant difference, with IOP at 30 days higher than at 7 days, while IOP at 90 days was significantly lower than at 30 days (*P* *=* .019) (Fig. 1C).

### 3.2 Differences of IOP between groups

IOP before surgery showed no statistically significant difference between emmetropic, mild to moderate myopic, and high myopic patients (*P* = .904). And no IOP difference was found at 1 and 7 days between 3 groups (*P* = .969 and *P* = .479, respectively). However, IOP at 30 days showed a great difference between groups (*P* = .013), with IOP at 30 days significantly higher in high myopia patients than that in emmetropic patients (*P* *=* .013) and mild to moderate myopic patients (*P* *=* .003). Interestingly, IOP were slightly higher in emmetropic group (12.7 ± 2.9) than other 2 groups (11.8 ± 2.8 and 11.4 ± 2.5, respectively) at 90 days, but it did not reach a statistically significance (*P* *=* .238) (Fig. 1E).

### 3.3 Differences of ΔIOP at each follow-up

Overall ΔIOP at 7 days (−2.1 ± 5.6) was significantly higher than that at 1 day after surgery (0.51 ± 5.7) (*P* < .001), ΔIOP at 30 days (−0.84 ± 3.8) was significantly lower than that at 7 days (*P* *=* .002), ΔIOP at 90 days (−0.71 ± 2.2) was lower than that at 30 days, while it did not reach a statistically significant level (*P* *=* .783), which indicated that IOP was decreasing relatively faster from 1 to 7 days than other follow-up period.

Emmetropic patients showed a significantly higher ΔIOP at 7 days (−2.1 ± 5.5) after surgery than that at 1 day (0.45 ± 5.6) (*P* = .013), and ΔIOP at 30 days (−0.79 ± 3.8) were lower than that at 7 days, ΔIOP at 90 days (−0.71 ± 2.6) were lower than that at 30 days, while they did not reach a statistically significant difference (*P* *=* .052 and *P* *=* .536, respectively).

Mild to moderate myopic patients, whose results were quite similar to that of emmetropic ones, showed a significantly higher ΔIOP at 7 days (−2.5 ± 5.7) than 1 day (0.57 ± 5.9) (*P* *=* .010). No significant difference of ΔIOP was observed at 30 days (−0.57 ± 2.7) compared with 7 days (*P* *=* .086) and at 90 days (−0.50 ± 1.9) compared with 30 days (*P* *=* .397).

**Figure 2:**
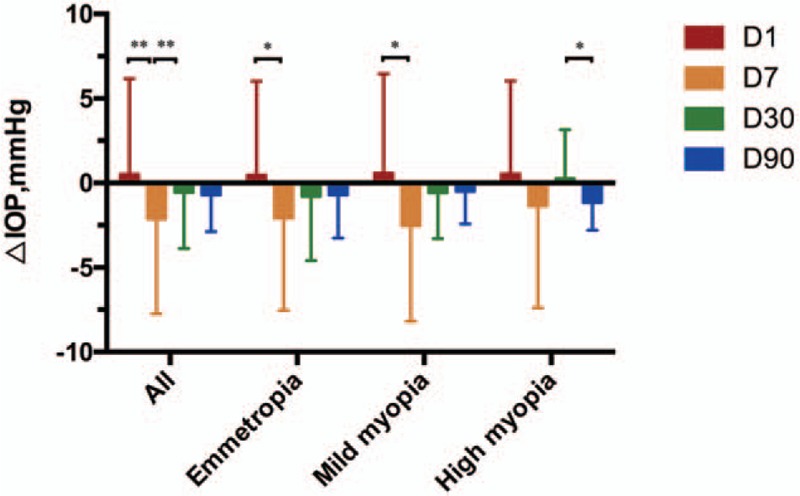
Differences of changes of intraocular pressure (ΔIOP) between groups. Mild myopia, mild to moderate myopia; D, days after operation. *P* < .05 compared with last follow-up was marked with ∗. *P* < .01 compared with last follow-up was marked with ∗∗. IOP = intraocular pressure.

However, high myopic patients showed no significant difference between ΔIOP at 7 days (−1.4 ± 6.0) and 1 day (0.52 ± 5.5) (*P* *=* .282), and between 30 days (0.26 ± 2.9) and 7 days (*P* *=* .116), while ΔIOP at 90 days (−1.2 ± 1.6) was significantly higher than that at 30 days, indicating that IOP was decreasing faster from 30 to 90 days in high myopic subjects. The results of ΔIOP are shown in Figure 2.

### 3.4 Total IOP changes at each follow-up between groups

Total IOP Change at 7 days after surgery is lower in high myopia patients than that in mild to moderate myopic patients (*P* *=* .022), while no significant difference was observed compared with emmetropic group (*P* = .110). And Total IOP Change at 30 days after surgery is significantly lower in high myopia patients than that in emmetropic patients (*P* = .013) and mild to moderate myopic patients (*P* = .005), and a notable difference between these 3 groups were found (*P* = .021), which may correspond to the IOP difference at 30 days in these groups. Although Total IOP Change at 90 days in emmetropia (−2.9 ± 2.9), mild to moderate myopia (−3.6 ± 2.9), and high myopia (−2.7 ± 1.5) showed no significant difference between groups (*P* = .238).

### 3.5 Subgroup analysis of patients receiving phacoemulsification

To evaluate the postoperative IOP influence of 2 cataract extraction methods (phaco and ECCE), subgroup analysis for factors mentioned above was conducted only for phaco. A total of 288 in 353 subjects received a phaco along with IOL implantation, among which 142 were emmetropia, 108 were mild to moderate myopia and 38 were high myopia. The cataract extraction methods were matched between 3 refractive conditions (*P* = .818).

The postoperative IOP changes were then analyzed in 3 refraction groups. For IOP at each follow-up, emmetropic patients showed a higher IOP at 1 day after surgery than that before surgery without significant difference (*P* = .476). IOP at 7, 30, and 90 days was significantly lower than that before surgery (all with *P* < .001), and IOP was significantly lower at 7 days compare with 1 day (*P* < .001), while no significant difference was found between 30 and 7 days as well as 90 and 30 days (*P* = .197 and *P* = .253, respectively). Similar to emmetropic patients, IOP at 1 day in mild to moderate myopia was higher than IOP before surgery with no significant difference (*P* = .209), and IOP at 7, 30, and 90 days were significantly lower than that before surgery (all with *P* < .001). IOP was significantly lower at 7 days compared with 1 day (*P* < .001), IOP at 30 days was lower than 7 days while IOP at 90 days higher compared with 30 days, both with no statistical significant difference (*P* = .193 and *P* *=* .163, respectively). In high myopia, we found a higher IOP at 1 day and lower IOP at 7 and 30 days compared with IOP before surgery with no statistic difference (*P* = .544, *P* *=* .677, and *P* *=* .163, respectively), along with a significantly lower IOP at 90 days than before surgery (*P* < .001). And no significant difference was found between 7 and 1 day, 30 and 7 days, and 90 and 30 days (*P* *=* .356, *P* *=* .829, and *P* *=* .432, respectively).

Same as the results of all patients, subgroup analysis revealed that IOP at 30 days showed a great difference between 3 groups (*P* = .030), while no difference was found at 1, 7, and 90 days (*P* = .153, *P* = .890, and *P* = .295, respectively). Based on the results above, it is reasonable to consider IOP changes in 3 refractive conditions the same between phaco and phaco/ECCE patients.

### 3.6 Relationship between IOP changes and basic characteristics

#### 3.6.1 Age

**Figure 3:**
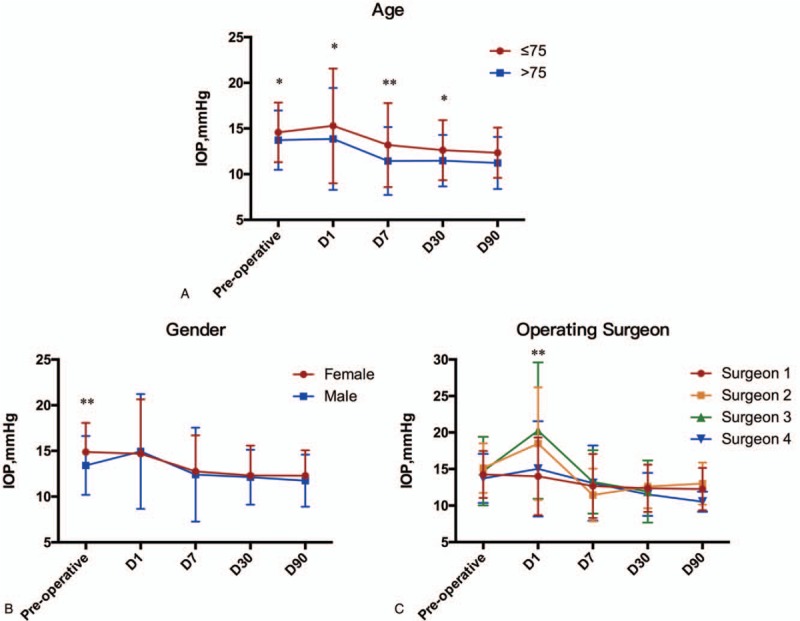
Different intraocular pressure at each follow-up divided by age and sex. (A) IOP at each follow-up before and after surgery in patients of different age. (B) IOP at each follow-up before and after surgery in male and female patients. (C) IOP at each follow-up before and after surgery in patients of different surgeon. D, days after operation. *P* < .05 between groups was marked with ∗. *P* < .01 between groups was marked with ∗∗. IOP = intraocular pressure.

Figure 3A shows the characteristics and IOP results by age. According to previous study,^[14]^ patients are stratified into 2 groups divided by the age of 75. Both groups showed an IOP increase with no statistic significance 1 day after surgery (*P* = .09 for group ≤75 and *P* = .715 for group >75), and revealed a significant IOP decrease in 7, 30, and 90 days compared with before cataract operation (all with *P* < .001). When we analyzed the IOP difference between these 2 groups, it is obvious that patients younger than 75 showed a significantly higher IOP than patients older than 75 before surgery (*P* = .020) as well as at 1, 7, and 30 days (*P* = .042, *P* = .002, and *P* = .021, respectively). Although the IOP at 90 days after surgery shows no significant difference between different ages (*P* = .131). As for Total IOP Changes, though there was a significant difference at 30 days between 2 ages, the Total IOP Changes at 7 and 90 days remained the same (*P* *=* .155 and *P* *=* .094, respectively).

#### 3.6.2 Gender

IOP results of different sex are shown in Figure 3B. Female patients (14.9 ± 3.2) showed a significantly higher IOP than male patients (13.4 ± 3.2) before surgery (*P* < .001). Interestingly, IOP of female patients decreased at 1 day after surgery than baseline, while it did not reach a significant difference (*P* = .670), and IOP at 7, 30, and 90 days was significantly lower than the IOP before surgery (all with *P* < .001). Male patients showed a significantly higher IOP at 1 day after surgery than that before surgery (*P* = .002), and IOP at 7, 30, and 90 days was significantly lower than the IOP before surgery (*P* = .010, *P* < .001, and *P* < .001, respectively). As for Total IOP Changes, women had a greater IOP reduction than men at 7 and 30 days (*P* = .033 and *P* = .015, respectively), which may be relevant to the IOP differences before surgery, while no significant difference was found at 90 days (*P* = .764). These results indicated that baseline IOP was different between male and female, with female IOP much higher than male patients, while the absolute value of IOP after surgery were not influenced by sex.

#### 3.6.3 Operating surgeon

IOP of patients between different surgeons showed a significant difference at 1 day after surgery (*P* < .001), even though IOP before surgery were statistically matched between surgeons (*P* = .230). The first day after surgery, patients of surgeon 1 (14.0 ± 5.3) showed a lower IOP than those of surgeon 2 (18.5 ± 7.7) and surgeon 3 (20.3 ± 9.3) (*P* < .001 and *P* = .011, respectively), and patients of surgeon 4 (15.0 ± 6.5) lower than those of surgeon 2 (*P* = .025). Patients of surgeon 1 showed a slightly lower IOP at 1 day after surgery than that before surgery (14.3 ± 3.2) with no significant difference (*P* = .448), while patients of surgeon 2 showed a significantly higher IOP at 1 day after surgery than that before surgery (*P* = .005) and no significant increase was found at 1 day after surgery with surgeon 3 and 4 (*P* = .108 and *P* = .160, respectively). Moreover, at 90 days after surgery, patients of surgeon 4 (10.5 ± 1.4) showed a lower IOP than those of surgeon 1 (12.3 ± 2.9) and surgeon 2 (13.0 ± 2.9) (*P* = .005 and *P* = .037, respectively) but not surgeon 3, as no patients of surgeon 3 finished the 90 days’ follow-up. All patients of different surgeons showed a significantly lower IOP at the last follow-up after surgery (at 90 days for surgeon 1, 2, and 4, and 30 days for surgeon 3) compared with before surgery (*P* < .001, *P* = .034, *P* = .011, and *P* = .007, respectively) and Total IOP Changes between the 4 surgeons at 7, 30, and 90 days showed no statistic difference (*P* = .179, *P* = .857, and *P* = .833, respectively). The results are shown in Figure 3C.

## 4 Discussion

In this study, we explored the relationship between refractive conditions and postsurgery IOP, and our results indicated that refractive conditions may influence the IOP fluctuation in first 90 days. However, all patients with different refractive conditions showed a significant IOP reduction at 90 days after surgery.

The IOP-lowering effect of cataract surgery has been long known in both glaucoma and non-glaucoma patients ranging from 3 to 60 months in previous studies,^[5,15]^ which was consistent with our study as all groups showed a significant lower IOP at 90 days compared with that before surgery, described in Figure 1. It is probably the result of the thin inserted IOL compared with the thickness of natural crystalline lens and that lens exchange deepens the anterior chamber.^[16,17]^

Transient IOP elevation in the early postoperative period after cataract surgery has been well described. Previous studies showed that IOP elevation may initiate at 2 hours with a maximum at 5 to 7 hours after surgery and last 24 hours typically.^[18,19]^ In a relevant retrospective cohort study, Bonnell et al reported a significant IOP elevation at 1 day after surgery, as 4.4% patients had an IOP elevation of 10 mm Hg and 2.1% had an IOP 30 mm Hg or higher after cataract surgery.^[20]^ In our study, IOP was found to be slightly elevated at 1 day after surgery, while it showed no statistically significant difference either in emmetropia or myopia patients. The probable reason of this phenomenon might be the inflammation reaction caused by surgery with blood-aqueous barrier abruption, and a blockage of the trabecular meshwork by remained lens cortical materials, viscoelastics, inflammation cell, and pigmented debris of iris.^[21,22]^

Refractive condition, represented by diopter value, has long been proved to be positively correlated with IOP measurements,^[23]^ with high myopia being a significant risk factor for primary open-angle glaucoma (POAG) development,^[24–26]^ and in patients with POAG, myopia contributed to IOP fluctuations as well as POAG progression.^[27]^ When it comes to the question whether refractive condition has the same effect on IOP changes after cataract surgery, 1 previous study observed no correlation between spherical refractive error and IOP at 3 to 6 month after cataract surgery,^[9]^ while no studies focused on the role of myopia in IOP in first 3 months after surgery. To our knowledge, our study is the first to specifically examine the relationship between refractive conditions and immediate IOP changes after cataract surgery.

In our study, patients with emmetropia and mild to moderate myopia showed exactly the same results. In these 2 groups, shown in Figure 1, IOP after surgery presented a significant decrease from 1 to 90 days after operation, especially at 1 to 7 days according to ΔIOP analysis according to Figure 2. However, in high myopia patients, no certain key decrease was observed from 1 to 30 days until a significant reduction was found from 30 to 90 days. This phenomenon indicated that high myopia may lead to a more smoothly IOP change in the early postoperational period and a relevantly delayed reduction compared with emmetropia and mild to moderate myopia patients.

The role of myopia in IOP changes after cataract surgery remains unclear. High myopia especially pathological myopia had several anatomic changes including increased visual axis length, retinal and choroidal thinning, enlarged optic disc, peripapillary atrophy, and insufficient hemoperfusions on choroid and the retina.^[11]^ In our study, though IOP before surgery was matched between groups, different patterns of IOP change after surgery were observed. We suspect that, especially in high myopia eyes, those anatomic changes mentioned above still existed and remained after cataract surgery, which might have an opposite contribution to the IOP reduction effect of cataract surgery. These changes might have already created more space in the anterior chamber, especially the long visual axis length and choroidal thinning along with thinner lens in high myopia patients, and therefore phaco with IOL implantation surgery may not have an instant effect on chamber angle, so the IOP reduction may not be immediately observed in those patients.

Interestingly, when comparing the IOP between 3 refractive conditions in each follow-up period, we found a significant difference at 30 days, with IOP higher in high myopia patients than that in emmetropic and mild to moderate myopic patients. Correspondingly, an IOP increase was identified from 7 to 30 days in high myopia group. We consider this phenomenon as a proof of the greater tendency of postoperative IOP fluctuation in high myopia patients, which was consistent with former studies.^[27]^

It is possible that the abnormal anatomic structures mentioned above in high myopia eyes, especially the insufficient hemoperfusions on choroid and retina, result in small amounts of autoregulation and lead to IOP fluctuation,^[27]^ which has been proved to be a risk factor for glaucoma progression.^[28]^ Previous study suggested that the irregular elevations of IOP may disrupt the homeostatic compensatory mechanisms in physiological IOP rhythmic cycles,^[29]^ and this was confirmed by an in vivo study concluding that fluctuations in IOP increased the density of the trabecular meshwork,^[30]^ which, to our knowledge, in turn lead to an IOP elevation and could play an opposite role in the IOP reduction effect of cataract surgery. Also these anatomical factors may induce pathological changes in retina and optic nerve, proved by a study showing that intermittent elevations in IOP produce changes in optic nerve consistent with early degeneration reported in chronic models of glaucoma.^[31]^ As an instability of postoperative IOP within 30 days was found in our study, we suggest that there should be a more frequent and longer follow-up for myopia, especially high myopia patients, and doctors should pay more attention to the IOP changes of these patients.

As for IOP changes, though Total IOP Changes at 7 and 30 days differed between groups, we found no significant difference with Total IOP Changes at 90 days, which proves that all the patients achieved a satisfactory IOP reduction through cataract surgery regardless of their refractive conditions. This might indicate that phaco with IOL implantation surgery played a more important role compared with structural features and the IOP fluctuation in high myopia eyes might finally came to be steady at 30 to 90 days after operation.

We also observed a younger age in myopic patients than control in our study (Fig. 3), which might indicate that myopia contributed to the younger onset of cataract. This result was also reported in previous studies as a complication of pathologic myopia,^[11]^ and suggested that a more frequent eye examination for patients with myopia was required for screening and early diagnosis of both cataract and other complications.

Previous study has found a small but statistically significant increase in IOP with age in western population.^[32,33]^ Although in several longitudinal studies focused on Asian population, IOP was found to be inversely associated with age.^[34,35]^ Same as the Asian studies mentioned above, our study reveals that the mean value of IOP before and after surgery is significantly higher in patients younger than 75 compared with patients older, and both achieved an effective postoperative IOP reduction. This may be explained by the reduced production of aqueous humor through aging,^[36]^ which leads to a reduction of IOP, and the structural changes in the trabecular meshwork,^[37]^ which increase IOP by increasing the resistance to aqueous humor outflow. The net change in IOP may be determined by the balance between these 2 processes, which may differ in Western and Asian populations. And our study proves that, despite the IOP change over aging, the final effect of phaco with IOL on IOP reduction is obvious in both age groups.

When analyzing IOP between different sex, a significantly higher IOP at baseline before surgery in female than that in male was observed. Previous studies have revealed a significantly higher IOP in female than male in young individuals,^[38]^ and IOP showed no significantly difference between premenopausal and postmenopausal women.^[39]^ The probable explanation of this phenomenon was the effects of sex hormones, as serum testosterone and estradiol were correlated with IOP.^[39,40]^ Meanwhile, IOP at each follow-up after surgery showed no significant difference between groups, which might indicate that cataract surgery played a more important role in IOP than sex differences.

We also found that, though IOP before surgery were matched between surgeons, IOP at 1 day after surgery varied in our study. Surgeon 1 showed a lower IOP at 1 day than that before surgery while other surgeons showed a higher one, and also the 1st-day postoperative IOP for surgeon 1 and 4 was relatively lower than surgeon 2 and 3. Results further reveal that at 90 days after cataract surgery, IOP of surgeon 4 was significantly lower than other surgeons. All patients of different surgeons reached a lower IOP at the last follow-up after surgery than before surgery, indicating that though different surgeons might influence the IOP fluctuation in first 3 months, the final effect of phaco with IOL implantation on IOP reduction remains steady. These results pointed that factors mentioned above might also be consequences of different performers.

Surgeons in our study were all well-trained professional cataract surgeons, including chief surgeon 1 who had completed more than 15,000 phaco with IOL implantation, attending surgeon 2 and 3 completed 5000 to 15,000, and resident surgeon 4 completed 2000 to 5000 operations. We noted that there is a smoother IOP fluctuation after surgery performed by the most experienced surgeon, and surprisingly, the least experienced one with even a significant lower IOP for surgeon 4 at 90 days compared with surgeon 1, 2, and 3. Former studies have concluded that there were a greater possibility of intraoperative complications and 1st-day postoperational IOP elevation in resident-performed phaco surgery compared with experienced surgeon;^[41–44]^ however, our study reveals a different outcome. We consider the higher carefulness of the less-experienced surgeon, which ensures a relatively better and thorougher management of viscoelastics, to be the main cause of this difference. The exclusion of several IOP-related complications in our study may as well contribute to this result and a selection of better patient conditions for residents should also be considered, as it had been suggested that patients with certain characteristics such as mature lenses and potential zonular pathology be assigned to more experienced surgeons.^[44]^ Also we assume that a single surgeon does not represent all, so further studies could be focused on increasing the number of surgeons of different experience with a balanced patient condition.

This study explores the relationship between postcataract surgery IOP and refractive conditions. A reduction of IOP in different refractive conditions was observed, and refractive conditions might influence the IOP fluctuation in first 90 days after surgery. Our study also has some limitations. First, there were patients from areas other than Beijing whose follow-up was performed in local hospitals, leading to some follow-up data lost. Second, the protocol does not include the evaluation of chamber angle configuration before and after surgery and there was a miss-recording of visual axis information in follow-system, which was matched by a paired test in each follow-up. Third, this study is a retrospective study, whose evidence is slightly lower than that in a prospective one. Future studies should focus on a prospective study on cataract surgery and IOP changes, and the role of cataract surgery in patients with a comorbidity of glaucoma and refractive errors requires more analysis.

In summary, a significant lower IOP at 90 days than that before surgery was found in emmetropic, mild to moderate myopic, and high myopic patients, which indicated that cataract surgery had an effect on IOP reduction despite the refractive conditions. However, high myopic patients showed a lower speed of IOP reduction with an instable IOP value in the first 30 days observed. Further studies should be focused on the relationship between anatomic changes of high myopic patients and their IOP after cataract surgery.

## Acknowledgment

The authors would like to thank Liyuan Tao for his statistical consultation.

## Author contributions

**Conceptualization:** Huibin Lv, Jiarui Yang, Xiaodan Jiang, Yan Liu, Mingzhou Zhang, Yuexin Wang, Hang Song, Xuemin Li.

**Data curation:** Huibin Lv, Jiarui Yang, Yuexin Wang.

**Formal analysis:** Jiarui Yang, Yushi Liu.

**Funding acquisition:** Xuemin Li.

**Investigation:** Huibin Lv, Jiarui Yang.

**Methodology:** Huibin Lv, Jiarui Yang, Yan Liu, Mingzhou Zhang, Yuexin Wang, Xuemin Li.

**Project administration:** Jiarui Yang, Xiaodan Jiang, Yan Liu, Mingzhou Zhang, Hang Song.

**Supervision:** Huibin Lv, Jiarui Yang, Xiaodan Jiang, Yan Liu, Mingzhou Zhang, Yuexin Wang, Hang Song, Xuemin Li.

**Validation:** Jiarui Yang.

**Visualization:** Yushi Liu.

**Writing – original draft:** Huibin Lv, Jiarui Yang, Yushi Liu.

**Writing – review & editing:** Huibin Lv, Jiarui Yang, Yushi Liu, Xiaodan Jiang, Yan Liu, Mingzhou Zhang, Yuexin Wang, Hang Song, Xuemin Li.

## References

[1] Pascolini D Mariotti SP Global estimates of visual impairment: 2010. Br J Ophthalmol 2012;96:614–8. 22133988 10.1136/bjophthalmol-2011-300539

[2] Liu YC Wilkins M Kim T Cataracts. Lancet 2017;390:600–12. 28242111 10.1016/S0140-6736(17)30544-5

[3] Fukuoka H Afshari NA The impact of age-related cataract on measures of frailty in an aging global population. Curr Opin Ophthalmol 2016;28:93–7. 10.1097/ICU.0000000000000338 27820747

[4] Javitt JC Brenner MH Curbow B Outcomes of cataract surgery: improvement in visual acuity and subjective visual function after surgery in the first, second, and both eyes. Arch Ophthalmol 1993;111:686–91. 8489454 10.1001/archopht.1993.01090050120041

[5] Melancia D Abegão PL Marques-Neves C Cataract surgery and intraocular pressure. Ophthalmic Res 2015;53:141–8. 25765255 10.1159/000377635

[6] Azuara-Blanco A Burr J Ramsay C Effectiveness of early lens extraction for the treatment of primary angle-closure glaucoma (EAGLE): a randomised controlled trial. Lancet 2016;388:1389–97. 27707497 10.1016/S0140-6736(16)30956-4

[7] Guan H Mick A Porco T Preoperative factors associated with IOP reduction after cataract surgery. Optometry Vision Sci 2013;90:179–84. 10.1097/OPX.0b013e31827ce224 23292045

[8] Yang HS Lee J Choi S Ocular biometric parameters associated with intraocular pressure reduction after cataract surgery in normal eyes. Am J Ophthalmol 2013;156:89–94. 23628350 10.1016/j.ajo.2013.02.003

[9] Slabaugh MA Chen PP The effect of cataract extraction on intraocular pressure. Curr Opin Ophthalmol 2014;25:122–6. 24463416 10.1097/ICU.0000000000000033

[10] Pan CW Ramamurthy D Saw SM Worldwide prevalence and risk factors for myopia. Ophthalmic Physiol Opt 2011;32:3–16. 10.1111/j.1475-1313.2011.00884.x 22150586

[11] Cho BJ Shin JY Yu HG Complications of pathologic myopia. Eye Contact Lens 2016;42:9–15. 26649982 10.1097/ICL.0000000000000223

[12] Nomura H Ando F Niino N The relationship between intraocular pressure and refractive error adjusting for age and central corneal thickness. Ophthalmic Physiol Opt 2004;24:41–5. 14687200 10.1046/j.1475-1313.2003.00158.x

[13] Ronald C Pruett MDPC Progressive myopia and intraocular pressure: what is the linkage?: a literature review. Acta Ophthalmol 1988;66:117–27. 2853515 10.1111/j.1755-3768.1988.tb02685.x

[14] Zetterström C Behndig A Kugelberg M Changes in intraocular pressure after cataract surgery: analysis of the Swedish National Cataract Register Data. J Cataract Refract Surg 2015;41:1725–9. 26432131 10.1016/j.jcrs.2014.12.054

[15] Jahn CE Reduced intraocular pressure after phacoemulsification and posterior chamber intraocular lens implantation. J Cataract Refract Surg 1997;23:1260–4. 9368174 10.1016/s0886-3350(97)80325-2

[16] Shin HC Subrayan V Tajunisah I Changes in anterior chamber depth and intraocular pressure after phacoemulsification in eyes with occludable angles. J Cataract Refract Surg 2010;36:1289–95. 20656150 10.1016/j.jcrs.2010.02.024

[17] Poley BJ Lindstrom RL Samuelson TW Intraocular pressure reduction after phacoemulsification with intraocular lens implantation in glaucomatous and nonglaucomatous eyes: evaluation of a causal relationship between the natural lens and open-angle glaucoma. J Cataract Refract Surg 2009;35:1946–55. 19878828 10.1016/j.jcrs.2009.05.061

[18] Bömer TG Lagrèze WD Funk J [Increased intraocular pressure after cataract extraction–effect of surgical technique, surgical procedure and preventive drug administration. A prospective, randomized double-blind study]. Klinische Monatsblätter Für Augenheilkunde 1995;206:13–9. 7897962 10.1055/s-2008-1035399

[19] Fang EN Kass MA Increased intraocular pressure after cataract surgery. Semin Ophthalmol 1994;9:235–42. 10155643 10.3109/08820539409060021

[20] Bonnell LN Soohoo JR Seibold LK One-day postoperative intraocular pressure spikes after phacoemulsification cataract surgery in patients taking tamsulosin. J Cataract Refract Surg 2016;42:1753–8. 28007106 10.1016/j.jcrs.2016.10.009

[21] Tennen DG Masket S Short-and long-term effect of clear corneal incisions on intraocular pressure. J Cataract Refract Surg 1996;22:568–70. 8784627 10.1016/s0886-3350(96)80010-1

[22] Morgan RK Skuta GL Viscoelastic-related glaucomas. Semin Ophthalmol 1994;9:229–34. 10155642 10.3109/08820539409060020

[23] David R Zangwill LM Tessler Z The correlation between intraocular pressure and refractive status. Arch Ophthalmol 1985;103:1812–5. 4074170 10.1001/archopht.1985.01050120046017

[24] Perdicchi A Iester M Scuderi G Visual field damage and progression in glaucomatous myopic eyes. Eur J Ophthalmol 2007;17:534–7. 17671927 10.1177/112067210701700409

[25] Buey MAD Lavilla L Ascaso FJ Assessment of corneal biomechanical properties and intraocular pressure in Myopic Spanish Healthy Population. J Ophthalmol 2014;2014:905129. 24719755 10.1155/2014/905129 PMC3955599

[26] Hsu CH Chen RI Lin SC Myopia and glaucoma: sorting out the difference. Curr Opin Ophthalmol 2015;26:90–5. 25565367 10.1097/ICU.0000000000000124

[27] Yang Y Li Z Wang N Intraocular pressure fluctuation in patients with primary open-angle glaucoma combined with high myopia. J Glaucoma 2014;23:19–22. 22668981 10.1097/IJG.0b013e31825afc9d

[28] Leidl MC Choi CJ Syed ZA Intraocular pressure fluctuation and glaucoma progression: what do we know? Br J Ophthalmol 2014;98:1315–9. 24627247 10.1136/bjophthalmol-2013-303980

[29] Caprioli J Coleman AL Intraocular pressure fluctuation: a risk factor for visual field progression at low intraocular pressures in the advanced glaucoma intervention study. Ophthalmology 2008;115:1123–9. 18082889 10.1016/j.ophtha.2007.10.031

[30] Zou H Yuan R Zheng Q Fluctuations in intraocular pressure increase the trabecular meshwork extracellular matrix. Cell Physiol Biochem 2014;33:1215–24. 24752241 10.1159/000358691

[31] Joos KM Li C Sappington RM Morphometric changes in the rat optic nerve following short-term intermittent elevations in intraocular pressure. Invest Ophthalmol Vis Sci 2010;51:6431–40. 20688743 10.1167/iovs.10-5212 PMC3055763

[32] Åström S Stenlund H Lindén C Intraocular pressure changes over 21 years-a longitudinal age-cohort study in northern Sweden. Acta Ophthalmol 2013;92:417–20. 23902137 10.1111/aos.12232

[33] Hennis A Wu SY Nemesure B Hypertension, diabetes, and longitudinal changes in intraocular pressure. Ophthalmology 2003;110:908–14. 12750088 10.1016/S0161-6420(03)00075-7

[34] Nakano T Tatemichi M Miura Y Long-term physiologic changes of intraocular pressure: a 10-year longitudinal analysis in young and middle-aged Japanese men. Ophthalmology 2005;112:609–16. 15808252 10.1016/j.ophtha.2004.10.046

[35] Zhao D Kim MH Pastor-Barriuso R A longitudinal study of age-related changes in intraocular pressure: the Kangbuk Samsung Health Study. Invest Ophthalmol Vis Sci 2014;55:6244–50. 25183763 10.1167/iovs.14-14151

[36] Brubaker RF Nagataki S Townsend DJ The effect of age on aqueous humor formation in man. Ophthalmology 1981;88:283–8. 7231919 10.1016/s0161-6420(81)35037-4

[37] Miyazaki M Segawa K Urakawa Y Age-related changes in the trabecular meshwork of the normal human eye. Jpn J Ophthalmol 1987;31:558–69. 3448324

[38] Dane S Aslankurt M Yazici AT Sex-related difference in intraocular pressure in healthy young subjects. Percept Motor Skill 2003;96:1314–6. 10.2466/pms.2003.96.3c.1314 12929788

[39] Ebeigbe JA Ebeigbe PN Sex hormone levels and intraocular pressure in postmenopausal Nigerian women. Afr J Med Med Sci 2013;42:317–23. 24839735

[40] Toker E Yenice O Temel A Influence of serum levels of sex hormones on intraocular pressure in menopausal women. J Glaucoma 2003;12:436–40. 14520153 10.1097/00061198-200310000-00007

[41] Elfersy AJ Prinzi RA Peracha ZH IOP elevation after cataract surgery: results for residents and senior staff at Henry Ford Health System. J Glaucoma 2016;25:802–6. 27027228 10.1097/IJG.0000000000000421

[42] Kim JY Jo MW Brauner SC Increased intraocular pressure on the first postoperative day following resident-performed cataract surgery. Eye 2011;25:929–36. 21527959 10.1038/eye.2011.93 PMC3178167

[43] Briszi A Prahs P Hillenkamp J Complication rate and risk factors for intraoperative complications in resident-performed phacoemulsification surgery. Graefes Arch Clin Exp Ophthalmol 2012;250:1315–20. 22527309 10.1007/s00417-012-2003-y

[44] Rutar T Porco TC Naseri A Risk factors for intraoperative complications in resident-performed phacoemulsification surgery. Ophthalmology 2009;116:431–6. 19167084 10.1016/j.ophtha.2008.10.028

